# Is single incision laparoscopic surgery (SILS) for gastric gastrointestinal stromal tumor (GIST) dependent on the location of the tumor?

**DOI:** 10.1186/s12893-023-02141-0

**Published:** 2023-08-21

**Authors:** Ji Won Seo, Ki Bum Park, Hyung Min Chin, Kyong-Hwa Jun

**Affiliations:** Department of Surgery, College of Medicine, St. Vincent’s Hospital, The Catholic University of Korea, Seoul, Republic of Korea

**Keywords:** Single-incision laparoscopic surgery, Laparoscopic surgery, Gastric wedge resection, GIST

## Abstract

**Background:**

We compared the surgical outcomes of single-incision laparoscopic surgery (SILS) and conventional laparoscopic surgery (CLS) for gastric gastrointestinal stromal tumor (GIST).

**Methods:**

We performed single-incision gastric wedge resection on prospectively-enrolled 15 consecutive patients with gastric GIST between November 2020 and April 2022 in a single tertiary center. The early perioperative outcomes of these patients were compared to those of patients who underwent CLS. The indications did not differ from those for conventional laparoscopic procedures for gastric GIST.

**Results:**

In total, 30 patients were assigned to the SILS (n = 15) and CLS (n = 15) groups. There were no significant differences in the estimated blood loss and intraoperative blood transfusion between the SILS and CLS groups. There were no intraoperative complications or conversions to multiple-port or open surgery in the SILS group. Proximally located tumors were more commonly treated with CLS than with SILS (*P* = 0.045). GISTs located in the greater curvature were more commonly treated with SILS than with CLS, although the difference was not statistically significant (*P* = 0.08). The mean incision length in the SILS group was 4.1 cm shorter than that in the CLS group (3.2 ± 0.7 and 7.3 ± 5.2 cm, respectively, *P* = 0.01). The postoperative analgesic dose was significantly lower in the SILS than in the CLS group (0.4 ± 1.4 and 2.1 ± 2.3, respectively *P* = 0.01). Also, the duration of postoperative use of analgesic was shorter in SILS than in CLS (0.4 ± 0.7 and 2.0 ± 1.8, respectively, *P* = 0.01). There were no significant differences in the early postoperative complications between the groups.

**Conclusions:**

SILS is as safe, feasible, and effective for the treatment of gastric GIST as CLS with comparable postoperative complications, pain, and cosmesis. Moreover, SILS can be considered without being affected by the type or location of the tumor.

**Supplementary Information:**

The online version contains supplementary material available at 10.1186/s12893-023-02141-0.

## Introduction

Gastrointestinal stromal tumors (GISTs) are rare stromal tumors of the gastrointestinal tract that have malignant potential [1]. Gastric GIST is the most common type, accounting for 70% of all GISTs [2]. GISTs smaller than 5 cm may be treated by laparoscopic resection performed by experienced surgeons if tumor free resection margins can be achieved, tumor capsule is preserved, and tumor spillage is avoided [3]. Gastric GIST has the best prognosis among all GISTs [4]. Traditionally, open resection was preferred by surgeons, but as the era of minimally invasive surgery started, laparoscopic resection has become the treatment of choice. Laparoscopic GIST wedge resection is comparable to open surgical resection in terms of the duration of surgery and complication rate. Moreover, it is associated with significantly less intraoperative blood loss, faster first flatus passage, earlier resumption of oral intake and mobilization and shorter hospital compared to open surgical resection [5].

Laparoscopic surgery has commonly been performed in the field of general surgery since the late 1980s [6]. Furthermore, laparoscopic gastrectomy was already proven to be a safe procedure with better short-term outcomes and recommended when performed by skillful surgeons [7]. The reduced number of ports in laparoscopic surgery has allowed the development of single-port laparoscopic surgeries. The first single-incision laparoscopic surgery (SILS) involved an appendectomy and was performed by Pelosi in 1992 [8]. SILS has become popular due to its acceptable clinical outcomes and good cosmetic outcomes [9]. In particular, single-port appendectomy, cholecystectomy, and bariatric surgery are performed worldwide [10–12]. There are a few sporadic reports of gastric GIST resection by SILS using special equipment and a small incision [13–15]. Wu et al. [16] and Takata et al. [17] also investigated several cases of single-port surgery for gastric submucosal tumor and proved the safety of SILS in 2013 and 2014. Afterwards, Kong et al. [18] identified 19 cases of gastric GIST treated using SILS and compared the outcomes of SILS and conventional laparoscopic surgery (CLS). However, they excluded tumors located at the cardia and pylorus [18]. To the best of our knowledge, the present study is the first to compare the surgical outcomes of SILS and CLS in prospectively-collected consecutive cases of gastric GIST, regardless of the tumor location.

In this study, we investigated the safety and feasibility of SILS in gastric GIST and compared the surgical outcomes including complications, cosmetic results, and use of analgesics between SILS and CLS. Additionally, we investigated whether SIILS for gastric GIST is dependent on the location of the tumor.

## Materials and methods

### Patients

**Fig. 1:**
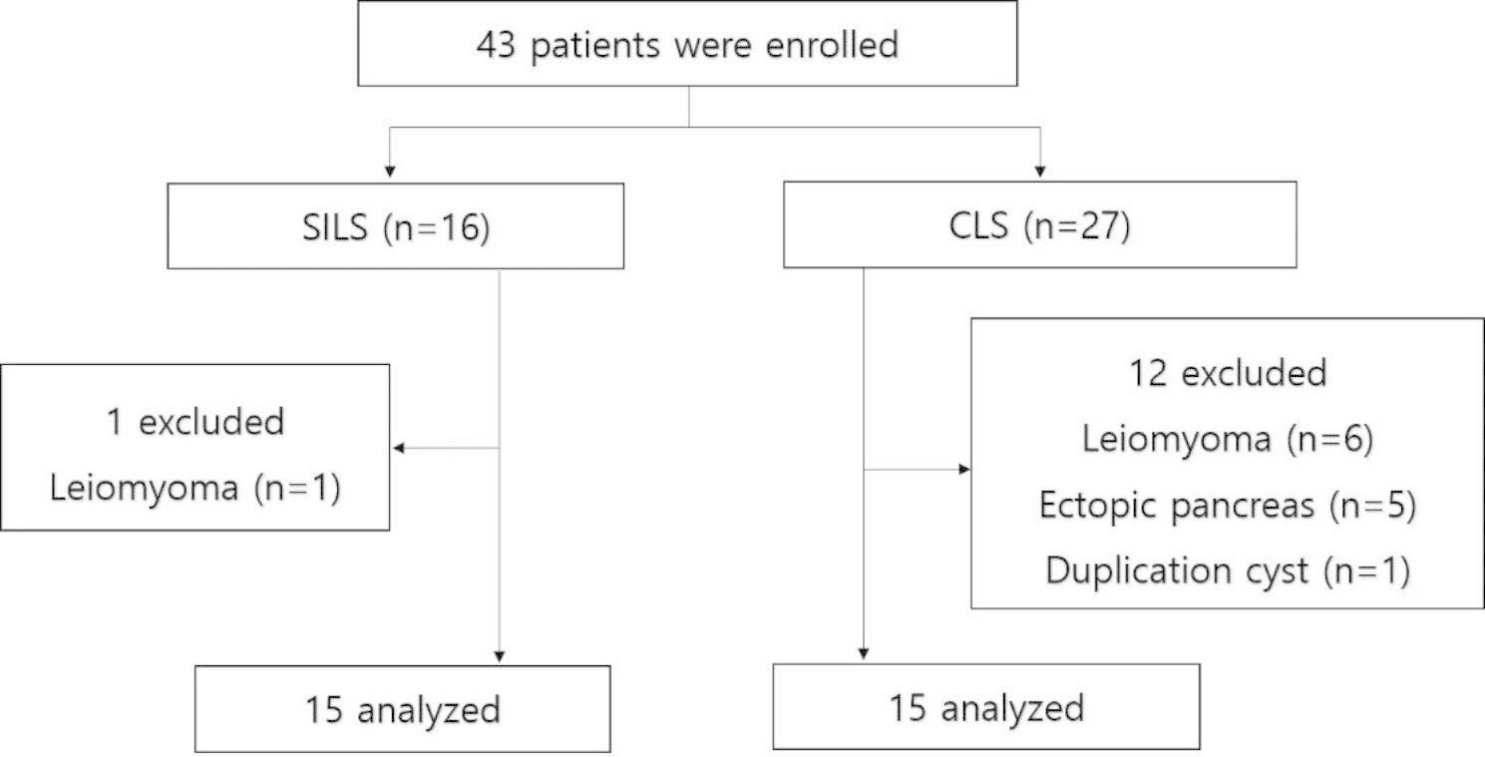
Flow chart for patient selection

This study was approved by the Institutional Review Board of St. Vincent’s Hospital, The Catholic University of Korea (VC23RASI0016). Informed consent was received from all patients. We analyzed the perioperative outcomes of single-incision gastric wedge resection procedure of 15 consecutive patients with gastric GIST between January 2020 and October 2022. Laparoscopic gastric wedge resection was performed for patients with gastric GISTs smaller than 5 cm regardless of their location. The indications for resection did not differ from those for CLS (Fig. 1). The surgical method was selected based on the surgeons’ preference. One surgeon (K.J.) performed SILS, whereas the other surgeons (H.C. and K.P.) performed CLS.

We collected the following patients’ demographic data: age, sex, American Society of Anesthesiologists classification (ASA), body mass index (BMI), history of abdominal surgery, and endoscopic and radiologic features of the tumor including size and location. The intraoperative findings included operative time, estimated blood loss, need for additional ports, conversion to open approach, and any postoperative complications. The postoperative outcomes included morbidity and mortality within 30 days of surgery, length of hospital stay, duration of pain, frequency of analgesic use, and time to initiation of soft diet. The postoperative morbidities were classified based on the Clavien-Dindo classification system, and pathology reports were reviewed.

### Single-incision laparoscopic gastric wedge resection procedure

A single surgeon performed all procedures. Under general anesthesia, the patient was placed in the supine reverse Trendelenburg position with both arms abducted. We made a 2.5- 3.0 cm long trans-umbilical incision through which a Glove Port device (Meditech Inframed Corp., Seoul, South Korea) containing a 10–15 mm port and three 3–10 mm ports was placed (Additional File Fig. 1). For tumors located near the esophagogastric junction (EGJ), the Glove Port device was inserted through the abdominal wall into the stomach and kept it in place by suspending the stomach from the anterior abdominal wall with sutures. At the end of the procedure, the gastric wall opening was closed using an endoscopic stapler. Pneumoperitoneum was achieved using carbon dioxide insufflation, and a continuous intra-abdominal pressure of 12 mmHg was maintained. The surgeon stood on the right side of the patient. The assistant stood on the left side of the patient, and the scopist stood between the legs of the patient. A 5- mm ENDOEYE FLEX scope (Olympus Medical Systems Corp., Tokyo, Japan), energy device (LigaSure™, Medtronic, Minneapolis, MN, USA), and grasper were inserted via the 3–10 mm port, whereas the stapler (Signia™ stapling system, Medtronic, Minneapolis, MN, USA) was inserted via the 10–15 mm port. A 45- mm or 60- mm endoscopic stapling device was used for gastric wedge resection after dividing the feeding vessels using an energy device. The umbilical incision was extended to a length of the tumor size, and the specimen was removed from the abdomen in a plastic bag. We did not routinely insert an intra-abdominal drain.

### Conventional laparoscopic gastric wedge resection procedure

In the CLS group, three to five ports were used. An 11- mm trocar was inserted through an infraumbilical incision, and pneumoperitoneum was established with carbon dioxide. A 12- mm trocar was inserted in the right mid- clavicular line, 2 cm above the umbilicus. The additional 5- mm trocars were then placed in both pre-axillary lines, 2 cm below the costal margin, or the left mid-clavicular line, 2 cm above the umbilicus. The operations were performed as described previously.

### Statistical analysis

Data were analyzed using SPSS software (ver. 21.0; IBM Corp., Armonk, NY, USA). Continuous variables are expressed as means ± standard deviations (SDs). Analysis of unpaired continuous variables was conducted using Student’s *t*-test. Paired continuous variables were analyzed using paired *t*-test. Categorical variables were compared using chi-squared test. The relationship between two variables was analyzed using Pearson correlation analysis. *P* < 0.05 was considered statistically significant. All tests were two-sided unless otherwise indicated.

## Results

### Patient characteristics

The demographics and pathological characteristics of the enrolled patients are summarized in Table 1. There were no significant differences in the baseline characteristics of patients, including age, sex, BMI, ASA score, and history of abdominal surgery between the SILS and CLS groups. Proximal tumor location was more common in the CLS group than in the SILS (*P* = 0.045). GISTs located in the greater curvature were more commonly treated with SILS than with CLS, although the difference was not statistically significant (*P* = 0.08). The mean tumor size was not different between the SILS and CLS (*P* = 0.41). Additionally, the risk of GIST according to the modified NIH classification was not different between the groups.

**Table 1:** Patient demographics and clinicopathological characteristics

Variable	SILS (n = 15)	CLS (n = 15)	*P* value
Age (years)	59.7 ± 12.6	58.6 ± 12.6	0.84
Sex Male Female	8 (57.1) 7 (42.9)	5 (33.3) 10 (66.7)	0.37
BMI (kg/m^2^)	24.7 ± 3.4	25.3 ± 3.8	0.71
ASA score I II	15 (100) 0	12 (80.0) 3 (20.0)	0.52
History of abdominal surgery No Yes	11 (71.4) 4 (28.6)	11 (73.3) 4 (26.7)	0.92
Tumor location Upper Middle Lower	4 (28.6) 11 (71.4) -	7 (46.7) 3 (20.0) 5 (33.3)	0.045
Gastric circumference Lesser curvature Greater curvature Anterior wall Posterior wall	2 (14.3) 11 (71.4) - 2 (14.3)	3 (20.0) 3 (20.0) 5 (33.3) 4 (26.7)	0.08
Location of gastric wall Submucosal Subserosal	4 (28.6) 11 (71.4)	7 (46.7) 8 (53.3)	0.33
Tumor size (cm)	3.2 ± 0.7	3.5 ± 1.3	0.41
Modified NIH classification Low Intermediate High	13 (85.7) 2 (14.3) -	8 (53.3) 6 (40.0) 1 (6.7)	0.32

Parentheses are percentage

### Postoperative clinical course and pain

As shown in Table 2, there were no significant differences in the estimated blood loss and intraoperative blood transfusions between the SILS and CLS groups. There were no intraoperative complications or conversions to multiple-port or open surgeries in the SILS group. However, there was one case of conversion to open surgery in the CLS group because of difficulty in the radical operation. The mean incision length in the SILS group was 4.1 cm shorter than that in the CLS group (3.2 ± 0.7 and 7.3 ± 5.2 cm, respectively, *P* = 0.01). The mean operation time was 12 min shorter in the SILS group than in the CLS group (66.7 ± 33.8 and 78.7 ± 35.4 min, respectively), however, the difference was not statistically significant (*P* = 0.46). There were no significant differences between the SILS and CLS groups in terms of the mean time until the first postoperative solid intake and postoperative hospital duration. Additionally, the inflammatory markers, including white blood cell count on the first day after surgery were not significantly different between the groups.

The postoperative analgesic doses were significantly lower in the SILS group than in the CLS (0.4 ± 1.4 and 2.1 ± 2.3, respectively, *P* = 0.01). Also, the duration of postoperative analgesic use was shorter in SILS than in CLS (0.4 ± 0.7 and 2.0 ± 1.8 days, respectively, *P* = 0.01).

**Table 2:** Intraoperative and postoperative findings

Variable	SILS (n = 15)	CLS (n = 15)	*P* value
Operation time (minutes)	66.7 ± 33.8	78.7 ± 35.4	0.46
Blood loss (mL)	2.5 ± 2.2	7.8 ± 12.5	0.28
Incision (cm)	3.2 ± 0.7	7.3 ± 5.2	0.01
Conversion To multiple port To open surgery	0 0	- 1	0.31
Transfusion	0	0	1.000
Time to first solid intake (days)	2.5 ± 1.7	3.0 ± 0.7	0.41
Frequencies of analgesic	0.4 ± 1.4	2.1 ± 2.3	0.01
Duration of use of analgesic (days)	0.4 ± 0.7	2.0 ± 1.8	0.01
Hospital stays (days)	6.1 ± 1.5	7.4 ± 3.8	0.28
White blood cell count, x10^3^/µl Preoperative Postoperative	5921 ± 1407.2 8705.7 ± 3646.2	5978 ± 1591.8 9599 ± 2415.6	0.937 0.57

Parentheses are percentage

### Postoperative intra-abdominal complications

Table 3 presents the surgical complications of the study patients. In the CLS group, two (13.3%) patients had early complications (one had anastomotic leakage, and the other had one wound infection). By contrast, in the SILS group, there were no early postoperative complications. One of the complications had a Clavien-Dindo grade II. None of the patients in the groups required reoperation or readmission. Furthermore, there were no cases of intraoperative or postoperative death.

**Table 3:** Postoperative intra-abdominal infectious complications

Variable	SILS (n = 15)		CLS (n = 15)		*P* value
Complication No Yes	15 (100) -	13 (86.7) 2 (13.3)		0.311	
Surgical complications Grade I Wound infection Grade II Leak	0 0	1 1			
Reoperation	0	0		1.00	
Readmission	0	0		1.00	
Postoperative mortality	0	0		1.00	

Parentheses are percentage

## Discussion

There were no significant differences in the intraoperative and postoperative complications between the SILS and CLS groups. Moreover, none of the patients who underwent SILS required open conversion or additional trocar insertion. Although the mean operation time was shorter in the SILS group than in the CLS group, the difference was not statistically significant. The frequency and duration of postoperative analgesic use was significantly lower in the SILS than in the CLS group. Therefore, SILS is safe and feasible for the treatment of gastric GIST. In the previous decade, improved knowledge of the biological behavior of GIST, as well as increased detection of this pathology has increased the interest in precise, segmental and mini-invasive resection techniques based on sound oncologic principles [19]. Importantly, lymph node dissection is not necessary because GIST rarely metastasizes to the lymph nodes. Moreover, the development of new operative tools and new operative techniques may improve the usefulness of SILS for gastric GIST. Therefore, SILS may become the preferred treatment for GIST in the future.

GISTs may develop in any part of the stomach [20]. The level of difficulty in resection depends on the location of gastric GISTs. Gastric anterior wall is easier to approach than the posterior wall, and the greater curvature is easier to approach than the lesser curvature. If the tumor is located on the posterior wall of the stomach, the gastrocolic omentum should be dissected, and the greater curvature should be mobilized to approach the lesion [21]. Moreover, it is difficult to use staplers on masses located at the posterior wall of the lesser curvature. In such cases, the tumor may be resected using laparoscopic ultrasound shears or a vascular sealing device instead of using a stapler that may result in deformation of the stomach and stenosis [22]. Finally, the proximal part is more difficult to access than the distal part. In particular, GISTs located near the EGJ are the most challenging to approach because EGJ narrowing may develop frequently after resection [23]. In other words, certain locations of GISTs may be more suitable for SILS. In the present study, SILS was performed less frequently for proximally located tumors and endophytic tumors, although the difference was not statistically significant. Preoperative imaging studies are required to select the appropriate surgical technique. Endoscopic ultrasonography and computed tomography are useful to determine the precise location of the GIST [24, 25]. In the present study, we performed preoperative endoscopic ultrasonography and abdominal computed tomography for both groups. The surgery was performed after the location of the gastric GIST was identified.

**Fig. 2:**
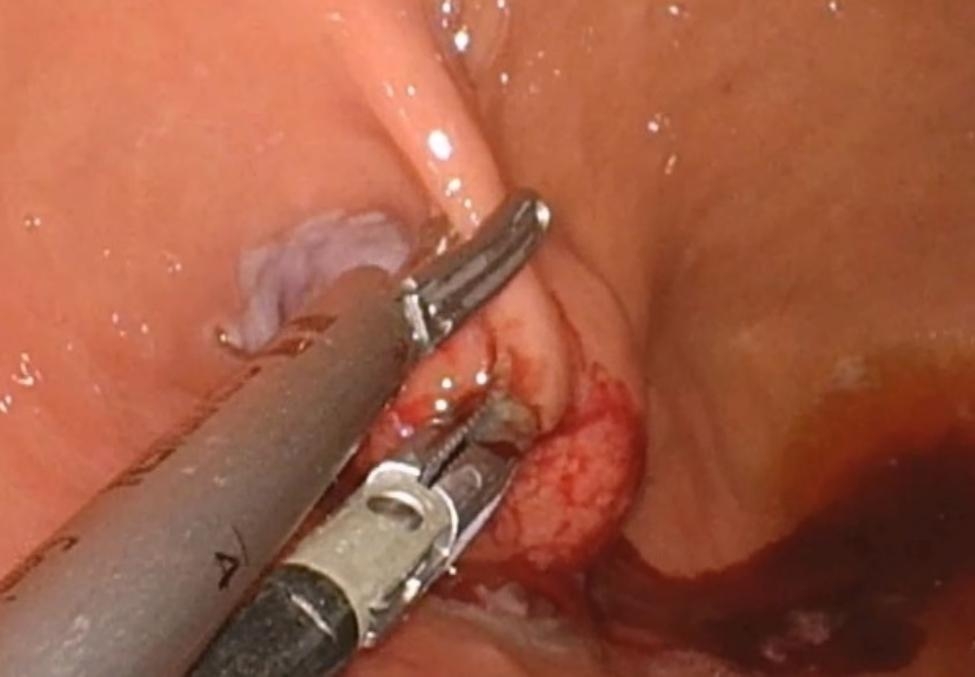
View of intragastric SILS

For GISTs located near the EGJ, extragastric approach is difficult because dissection along the greater curvature by ligating the short gastric vessels near the spleen is necessary [26]. Similarly, extragastric approach is complicated when endophytic GIST is located in the cardia or high body. Tumor identification and localization solely by visual exploration and palpation of the abdominal cavity during laparoscopy is not always successful [27]. Laparoscopic intraluminal (intragastric) surgery is useful to resect endophytic GISTs located near the EGJ (Fig. 2). In this approach, first described by Ohashi in 1995, the laparoscopic ports are passed through the abdominal wall, and then the gastric wall, into the gastric cavity [28]. When the intragastric approach is performed in cases of endophytic GISTs located in the cardia, multiple gastric holes are required to reach the gastric cavity for CLS. By contrast, a single gastric incision is required for SILS, which avoids the need for multiple gastric wall defects. A Glove Port is inserted through the incisional site on the anterior wall of the stomach and maintained in place by suspending the stomach from the anterior abdominal wall using sutures. In the present study, two intragastric surgeries were performed. An endoscopic stapler was used for resection in a single case, whereas in another case, the mass was resected using an energy device. In both cases, subsequent barbed suturing was performed. After suturing, an indocyanine green leak test was performed. There were no conversions to multiple-port or open surgeries in the intragastric SILS group in this study. Additionally, no EGJ stenosis was observed in either case in follow-up endoscopy. Intragastric approach makes patients with endophytic GIST near the cardia be undergone SILS safely.

The size of the submucosal tumor plays an important role in the selection of the operative technique. For wedge resection using a linear stapler, the radius of the resected gastric wall must be 2π-fold greater than the radius of the tumor. Therefore, the gastric wall defect should be 3-fold larger than the tumor size. For large tumors, this may cause deformation of the stomach and stenosis [29]. In the present study, the mean tumor size was not different between the SILS and CLS groups, and did not exceed 5 cm. Lee et al. [30] proposed a decision-making algorithm based on tumor size, location, and growth pattern using receiver operating characteristic curve analysis and multivariate logistic regression of 57 patients with submucosal tumor treated using laparoscopic and endoscopic cooperative techniques. In the present study, the size, location, and growth pattern of gastric GIST influenced the outcomes of SILS although multivariate analysis was not performed due to the small sample size.

The greatest advantage of minimally invasive surgery is the smaller incision compared to open surgery. Previous meta-analyses and randomized controlled trials have reported better cosmetic results and patient satisfaction with minimally invasive surgery compared to open surgery [31, 32]. Although trocars used for multiple port laparoscopic surgery have a size ≤ 12 mm, the sum of the length of the trocar sites are longer than that for SILS. In the present study, the length of the SILS incision was shorter than the sum of incision lengths for the conventional laparoscopic surgery. Most surgeons use an umbilical incision for SILS. As a result, postoperative wounds are barely visible because of the umbilical dimple. Deveci et al. [33] found that SILS was associated with improved cosmetic satisfaction compared to conventional laparoscopic surgery. Similarly, in the present study, the SILS wound was barely noticeable, leading to improved cosmetic results (Additional File Fig. 2).

The use of analgesics was significantly less common in the SILS group than in the CLS group. Moreover, the duration of use of analgesics was shorter in the SILS group than in the CLS group, indicating less postoperative pain in the former. Postoperative pain depends on several factors including the length of incision, number of ports, and individual sensitivity to pain [34]. Fewer incisions in the abdominal wall may lead to decreased parietal pain. Incision sites are painful due to trauma and fascial tension created by surgical closure [35]. Omori et al. [35] also found that the 5- mm and 12- mm trocars produced constant pain and that differences in trocar diameter did not affect postoperative pain. In the present study, pain in the upper abdomen, where no incision was made, might have been less in the SILS group than in the CLS group, leading to less frequent and shorter duration of analgesic use. Moreover, Bulut et al. [36] found that the median levels of C-reactive protein were significantly lower in a single-port surgery group than in a multiple-port surgery group, indicating that the former had positive effects on the acute phase response to trauma-induced immunomodulation. By contrast, postoperative white blood cell count was lower in the SILS group than in the CLS group, although the difference was not statistically significant.

Although this study was the surgeon’s initial experience with SILS for gastric GIST, there were no significant differences in postoperative complications between the SILS and CLS groups. In the CLS group, two patients experienced surgical complications. However, none of the patients in the SILS group experienced any complications. Previous studies of gastric GISTs have found that the complication rate is lower for SILS than for CLS [37, 38]. These results suggest that SILS is safe and feasible for the treatment of GIST. Additionally, Kong et al. [18] found that the operative time was shorter for an SILS group than for a conventional group. In the present study, the mean operation time was shorter in the SILS group than in the CLS group, although the difference was not statistically significant. The operation time for SILS have been reduced because the operator had previously performed multiple-port laparoscopic gastric wedge resections. SILS was only performed once the operator had overcome the learning curve. Therefore, the surgical outcomes of SILS may be better than those of CLS. Moreover, the laparoscopic and endoscopic rendezvous and the suture-lesion-lifting methods for the resection of gastric GISTs may assist novice operators to overcome the obstacles in learning SILS even when one has no experience in performing these combination methods [17, 39]. Future studies should perform intraoperative endoscopy with SILS to accurately identify the tumor location and confirm the absence of postoperative bleeding and leakage.

This study had certain limitations. First, our results have limited generalizability because of the small sample size. We only investigated the outcomes of 30 patients. Future studies should include a larger sample size. Second, we could not exclude the presence of selection bias. We did not use randomization to select the surgical technique. Future studies are needed to validate the selection criteria for SILS and CLS. Since this study is a pilot study, we plan to investigate a randomized controlled trial with a bigger sample in the future. Finally, we did not use the visual analogue scale score to assess postoperative pain. Further studies should use numeric scores to evaluate postoperative pain.

In conclusion, SILS is safe, feasible, and effective for the treatment of gastric GIST, and has comparable postoperative complications, pain, and cosmesis to CLS. Moreover, SILS can be considered without being affected by the type or location of the tumor. SILS may be an alternative for the treatment of gastric GIST. Further studies with longer follow-up are needed to determine if SILS is superior to CLS for the treatment of gastric GISTs.

### Electronic supplementary material

Below is the link to the electronic supplementary material.

**Additional File Fig. 1:** Port placement of single port laparoscopic gastric wedge resection.

**Additional File Fig. 2:** Postoperative view of the patient with single port laparoscopic gastric wedge resection.

## Data Availability

The datasets generated during the current study are not publicly available due to our institution’s policy, but are available from the corresponding author on reasonable request.
