# Supplementary material for: Is single incision laparoscopic surgery (SILS) for gastric gastrointestinal stromal tumor (GIST) dependent on the location of the tumor?
Source: BMC Surg. 2023 Aug 21;23:247. doi: 10.1186/s12893-023-02141-0 (PMC10441706; doi:10.1186/s12893-023-02141-0)
Supplement: Supplementary file 1 — Additional File Fig. 1: Port placement of single port laparoscopic gastric wedge resection. [file 12893_2023_2141_MOESM1_ESM.docx]

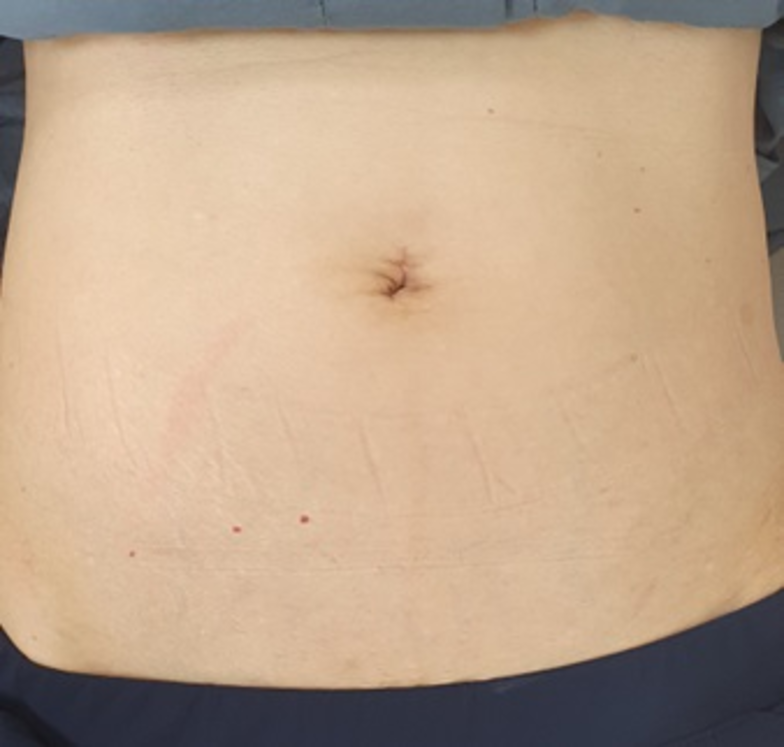


**Supplemental figure 2.** Postoperative view of the patient with single port laparoscopic gastric wedge resection.
