# Supplementary figures and images for: Is single incision laparoscopic surgery (SILS) for gastric gastrointestinal stromal tumor (GIST) dependent on the location of the tumor?
Source: BMC Surg. 2023 Aug 21;23:247. doi: 10.1186/s12893-023-02141-0 (PMC10441706; doi:10.1186/s12893-023-02141-0)

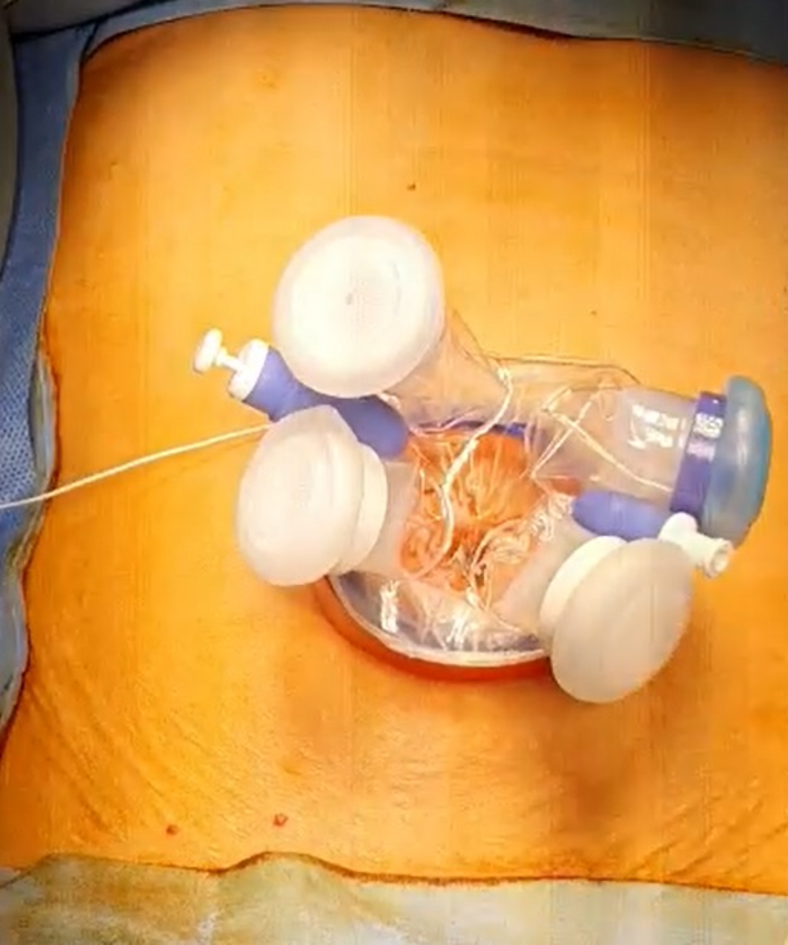


**Supplemental figure 1.** Port placement of single port laparoscopic gastric wedge resection.

Supplement: Supplementary file 2 — Additional File Fig. 2: Postoperative view of the patient with single port laparoscopic gastric wedge resection. [file 12893_2023_2141_MOESM2_ESM.docx]
